# Supplementary material for: Individual differences in knowledge network navigation
Source: Sci Rep. 2024 Apr 9;14:8331. doi: 10.1038/s41598-024-58305-2 (PMC11379931; doi:10.1038/s41598-024-58305-2)
Supplement: Supplementary file 2 — Supplementary Information 2. [file 41598_2024_58305_MOESM2_ESM.pdf]

Supplementary Figures

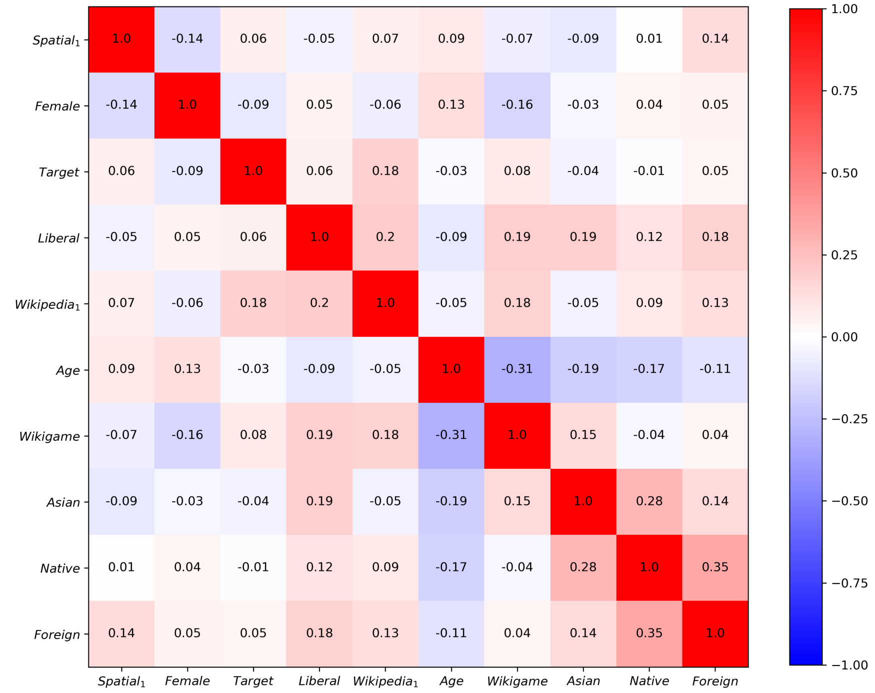

Supplementary Figure 1. **Correlation of the significant predictors of the navigation performance**

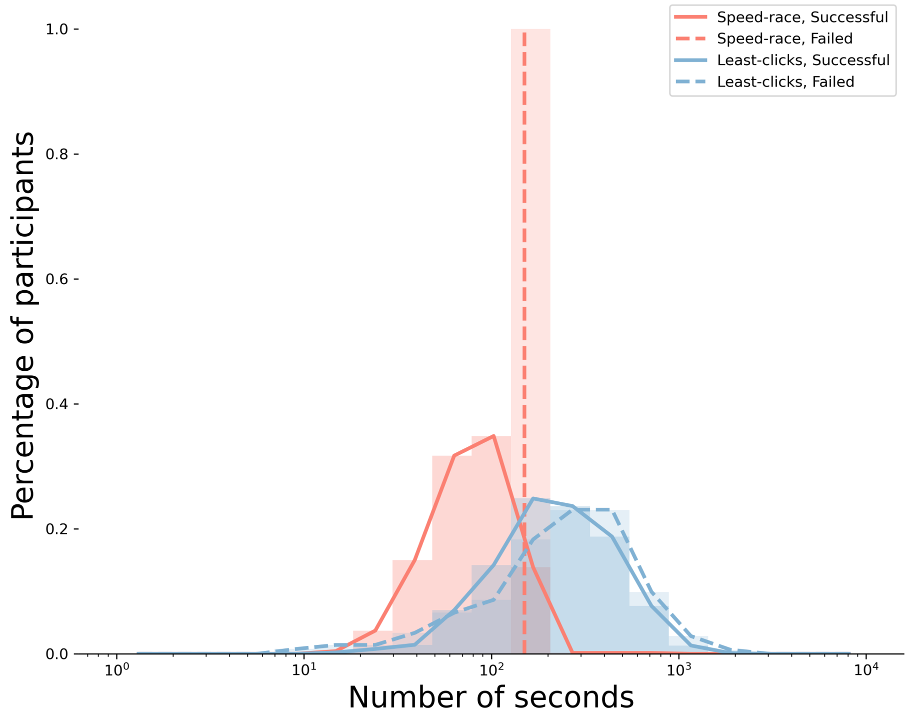

Supplementary Figure 2. Distribution of the time spent in each game This figure shows the distribution of time (in seconds) spent in the successful and failed Speed-race games and Least-clicks games respectively. For the Speed-race games, the median game time for the successful and failed games are 78 seconds and 150 seconds (the time limit for the game), and for Least-clicks games 214 seconds and 249 seconds.

Supplementary Tables

Supplementary Table S1. **Description characteristics of the study group.**

Characteristic	Female (N = 203)		Male (N = 192)		Total (N = 397)	
**Age**						
Mean ± S.D.	34.6 ± 11.1		32.0 ± 9.6		33.2 ± 10.5	
Minimum	19		19		19	
Maximum	64		77		77	
**Political Position (N, %)**						
Liberal	125	(61.6)	109	(56.8)	235	(59.2)
Moderate	41	(20.2)	50	(26.0)	91	(22.9)
Conservative	25	(12.3)	21	(10.9)	47	(11.8)
Other	12	(5.9)	12	(6.2)	24	(6.0)
**Ethnicity Background (N, %)**						
White	103	(50.7)	81	(42.2)	185	(46.6)
African	36	(17.7)	38	(19.8)	74	(18.6)
Asian	34	(16.7)	37	(19.3)	71	(17.9)
Hispanic	27	(13.3)	35	(18.2)	63	(15.9)
Other	3	(1.5)	1	(0.5)	4	(1.0)
**Language (Speaks a foreign language) (N, %)**						
No	98	(48.3)	103	(53.6)	201	(50.6)
Yes	105	(51.7)	89	(46.4)	196	(49.4)
**Language (Has a foreign native language) (N, %)**						
No	164	(80.8)	161	(83.9)	327	(82.4)
Yes	39	(19.2)	31	(16.1)	70	(17.6)
**Big Five Personality Traits (Mean ± S.D.)**						
Agreeableness	20.1 ± 4.6		20.7 ± 4.1		20.3 ± 4.4	
Conscientiousness	20.0 ± 4.5		20.1 ± 4.4		20.0 ± 4.5	
Extraversion	20.0 ± 4.6		20.0 ± 4.4		20.0 ± 4.5	
Neuroticism	19.3 ± 4.6		19.3 ± 4.6		19.4 ± 4.6	
Openness to Experience	21.0 ± 4.5		20.3 ± 4.1		20.6 ± 4.3	
**Prior Experience with the Wikigame (N, %)**						
I have never heard of the game and never played it before	157	(77.3)	119	(62.0)	278	(70.0)
I have heard of the game but never played it before	35	(17.2)	51	(26.6)	86	(21.7)
I have played the game (or similar game) several times before	10	(4.9)	15	(7.8)	25	(6.3)
I have played the game (or similar game) many times before	1	(0.5)	4	(2.1)	5	(1.3)
Other	0	(0)	3	(1.6)	3	(0.8)
**Number of Games Won**						
Mean ± S.D.	4.0 ± 2.6		4.4 ± 2.6		4.2 ± 2.6	
**Number of Games Played in Each Type (Mean ± S.D.)**						
Speed-race (game with time constraint)	3.8 ± 3.3		4.7 ± 3.3		4.3 ± 3.3	
Least-clicks (game with distance constraint)	5.2 ± 3.3		4.3 ± 3.3		4.7 ± 3.3	

Supplementary Table S2. **Logistic regression results for the success of the navigation routes in the Speed-race games.**

	Dependent variable: Success								
	Speed-race Games								
	(1)	(2)	(3)	(4)	(5)	(6)	(7)	(8)	(9)
Age				-0.046^{***}	-0.043^{***}	-0.046^{***}	-0.045^{***}	-0.046^{***}	-0.046^{***}
				(0.007)	(0.007)	(0.007)	(0.007)	(0.007)	(0.008)
Female									-0.217
									(0.121)
Asian American					0.711^{***}	0.791^{***}	0.829^{***}	0.963^{***}	0.942^{***}
					(0.156)	(0.159)	(0.160)	(0.168)	(0.169)
Foreign Language (Native)								-0.501^{**}	-0.493^{**}
								(0.179)	(0.179)
Foreign Language			0.921^{***}	0.802^{***}	0.740^{***}	0.700^{***}	0.662^{***}	0.767^{***}	0.784^{***}
			(0.115)	(0.117)	(0.119)	(0.120)	(0.121)	(0.127)	(0.127)
Wikipedia_1							0.181^{***}	0.206^{***}	0.204^{***}
							(0.053)	(0.054)	(0.054)
Prior (Wikigame)		0.988^{***}	1.019^{***}	0.824^{***}	0.777^{***}	0.739^{***}	0.687^{***}	0.640^{***}	0.621^{***}
		(0.089)	(0.092)	(0.096)	(0.096)	(0.097)	(0.098)	(0.099)	(0.100)
Prior (Target Page)						0.245^{***}	0.205^{***}	0.200^{***}	0.189^{**}
						(0.058)	(0.059)	(0.059)	(0.059)
Order	0.071^{***}	0.081^{***}	0.085^{***}	0.089^{***}	0.091^{***}	0.091^{***}	0.093^{***}	0.096^{***}	0.096^{***}
	(0.020)	(0.021)	(0.021)	(0.022)	(0.022)	(0.022)	(0.022)	(0.022)	(0.022)
Constant	-1.038^{***}	-1.634^{***}	-2.174^{***}	-0.612	-0.782^{*}	-1.152^{***}	-1.114^{**}	-1.038^{**}	-0.928^{**}
	(0.181)	(0.199)	(0.217)	(0.319)	(0.325)	(0.341)	(0.343)	(0.346)	(0.351)
Observations	1,479	1,479	1,479	1,479	1,479	1,479	1,479	1,479	1,479
Deviance	1999.427	1858.119	1791.641	1746.891	1725.745	1707.355	1695.463	1687.544	1684.359
Log Likelihood	−999.713	−929.059	−895.821	−873.445	−862.873	−853.678	−847.731	−843.772	−842.180
Akaike Inf. Crit.	2,017.427	1,878.119	1,813.641	1,770.891	1,751.745	1,735.355	1,725.463	1,719.544	1,718.359
Note:									*p<0.05; **p<0.01; ***p<0.001

Supplementary Table S3. **Logistic regression results for the success of the navigation routes in the Least-clicks games.**

	Dependent variable:Success							
	Least-clicks Games							
	(1)	(2)	(3)	(4)	(5)	(6)	(7)	(8)
Age						-0.017^{***}	-0.019^{***}	-0.020^{***}
						(0.005)	(0.005)	(0.005)
Foreign Language				0.517^{***}	0.450^{***}	0.438^{***}	0.408^{***}	0.385^{***}
				(0.102)	(0.104)	(0.104)	(0.105)	(0.106)
Liberal					0.431^{***}	0.442^{***}	0.464^{***}	0.435^{***}
					(0.106)	(0.106)	(0.107)	(0.108)
Wikipedia_1								0.098^{*}
								(0.044)
Spatial_1							0.128^{**}	0.123^{**}
							(0.044)	(0.044)
Prior (Wikigame)		0.611^{***}	0.579^{***}	0.576^{***}	0.511^{***}	0.421^{***}	0.429^{***}	0.394^{***}
		(0.096)	(0.098)	(0.099)	(0.100)	(0.102)	(0.102)	(0.103)
Prior (Target Page)			0.277^{***}	0.276^{***}	0.276^{***}	0.270^{***}	0.260^{***}	0.239^{***}
			(0.048)	(0.048)	(0.049)	(0.049)	(0.049)	(0.050)
Order	0.020	0.021	0.018	0.018	0.018	0.018	0.018	0.019
	(0.018)	(0.018)	(0.019)	(0.019)	(0.019)	(0.019)	(0.019)	(0.019)
Constant	−0.136	−0.297	-0.760^{***}	-1.006^{***}	-1.214^{***}	-0.602^{*}	−0.511	−0.400
	(0.156)	(0.160)	(0.181)	(0.189)	(0.197)	(0.263)	(0.265)	(0.270)
Observations	1,662	1,662	1,662	1,662	1,662	1,662	1,662	1,662
Deviance	2275.619	2231.278	2197.145	2171.371	2154.814	2142.421	2133.731	2128.874
Log Likelihood	−1,137.809	−1,115.639	−1,098.573	−1,085.685	−1,077.407	−1,071.211	−1,066.866	−1,064.437
Akaike Inf. Crit.	2,293.619	2,251.278	2,219.145	2,195.371	2,180.814	2,170.421	2,163.731	2,160.874

Note: *p<0.05; **p<0.01; ***p<0.001

Supplementary Table S4. Regression results. The table shows the regression results for navigation performance in the Speed-race games and Least-clicks games, where interactions are excluded or included in the model. The interaction terms are chosen in the following way: firstly, from all the pairwise interaction terms we selected those that are significant predictors of the players' navigation performance ($p < 0.01$) and do not have colinearity issues (VIF < 5) when added alone to the logistic regression model without interaction terms. Then we add all the selected interaction terms to the logistic regression model and filtered out those that are not significant ($p < 0.01$) at predicting the players' navigation performance. The interaction terms chosen this way are shown in the table for the Speed-race games and Least-clicks games respectively.

	Dependent variable: navigation performance			
	Speed-race Games		*Least-clicks Games*	
	Without Interactions	With Interactions	Without Interactions	With Interactions
Age	−0.053*** (0.008)	−0.062*** (0.009)	−0.021*** (0.006)	−0.024*** (0.006)
Female	−0.362** (0.136)	−0.405** (0.141)	0.086 (0.118)	0.102 (0.120)
Asian American	0.812*** (0.181)	0.585** (0.189)	0.168 (0.159)	0.200 (0.163)
African American	−0.452* (0.186)	−0.505** (0.193)	−0.293* (0.138)	−0.223 (0.139)
Foreign Language (Native)	−0.601** (0.190)	−0.524** (0.194)	−0.130 (0.155)	−0.182 (0.158)
Foreign Language	0.721*** (0.141)	0.665*** (0.146)	0.457*** (0.120)	0.473*** (0.122)
Liberal	0.086 (0.137)	−0.062 (0.145)	0.411*** (0.117)	0.415*** (0.119)
Agreeableness	−0.012 (0.015)	−0.017 (0.015)	−0.002 (0.013)	−0.010 (0.013)
Conscientiousness	0.026 (0.015)	0.036* (0.016)	0.020 (0.013)	0.012 (0.013)
Extroversion	−0.031* (0.014)	−0.027 (0.015)	−0.013 (0.013)	−0.013 (0.013)
Neuroticism	−0.007 (0.014)	−0.001 (0.015)	0.021 (0.012)	0.023 (0.012)
Openness	0.018 (0.015)	0.032* (0.016)	−0.017 (0.013)	−0.021 (0.013)
Wikipedia$_1$	0.206*** (0.060)	0.223*** (0.063)	0.130** (0.050)	0.126* (0.050)
Wikipedia$_2$	0.081 (0.085)	−0.074 (0.097)	−0.108 (0.078)	−0.111 (0.079)
Spatial$_1$	0.097 (0.053)	0.123* (0.054)	0.160*** (0.048)	0.170*** (0.048)
Spatial$_2$	0.004 (0.059)	−0.047 (0.061)	0.067 (0.057)	0.073 (0.058)
Spatial$_3$	0.081 (0.068)	0.091 (0.072)	−0.111* (0.053)	−0.113* (0.054)
Spatial$_4$	−0.030 (0.075)	0.065 (0.078)	0.120 (0.065)	0.008 (0.071)
Employment$_1$	0.033 (0.048)	−0.049 (0.054)	−0.014 (0.042)	0.031 (0.045)
Employment$_2$	−0.045 (0.058)	−0.092 (0.060)	−0.107* (0.049)	−0.105* (0.049)
Employment$_3$	−0.116 (0.079)	−0.475*** (0.144)	−0.023 (0.067)	−0.049 (0.068)
Education$_1$	0.063 (0.060)	0.066 (0.062)	−0.083 (0.048)	−0.065 (0.049)
Education$_2$	−0.047 (0.098)	−0.058 (0.106)	−0.023 (0.081)	−0.043 (0.083)
Computer$_1$	−0.102* (0.047)	−0.169*** (0.050)	0.051 (0.045)	0.031 (0.046)
Computer$_2$	−0.020 (0.084)	−0.038 (0.087)	−0.072 (0.077)	−0.122 (0.078)
Prior (Wikigame)	0.562*** (0.107)	0.712*** (0.113)	0.394*** (0.110)	0.371** (0.115)
Prior (Source Page)	−0.087 (0.068)	−0.094 (0.071)	−0.021 (0.057)	−0.010 (0.058)
Prior (Target Page)	0.192** (0.068)	0.222** (0.071)	0.264*** (0.055)	0.280*** (0.056)
Order	0.103*** (0.023)	0.107*** (0.023)	0.020 (0.019)	0.016 (0.020)
Game Index A1	1.059*** (0.251)	1.107*** (0.258)	0.442* (0.221)	0.458* (0.223)
Game Index A2	0.746** (0.263)	0.814** (0.270)	0.389 (0.232)	0.449 (0.235)
Game Index B0	1.598*** (0.262)	1.757*** (0.270)	0.575** (0.214)	0.616** (0.216)
Game Index B1	0.947*** (0.264)	1.033*** (0.272)	−0.053 (0.218)	−0.026 (0.220)
Game Index B2	0.528* (0.254)	0.603* (0.261)	−0.101 (0.219)	−0.081 (0.221)
Game Index O1	0.757** (0.247)	0.823** (0.253)	0.115 (0.212)	0.128 (0.214)
Game Index O2	0.658* (0.276)	0.742** (0.285)	0.316 (0.238)	0.334 (0.240)
African American:Employment$_1$		0.401** (0.126)		
Foreign Language (Native):Wikipedia$_2$		0.762*** (0.225)		
Liberal:Employment$_3$		0.553** (0.181)		
Wikipedia$_1$:Employment$_1$		0.209*** (0.038)		
Wikipedia$_2$:Computer$_2$		0.391** (0.119)		
Asian American:Employment$_1$				−0.302** (0.112)
Wikipedia$_2$:Education$_2$				0.318** (0.116)
Spatial$_4$:Prior (Wikigame)				0.463*** (0.127)
Constant	−0.192 (0.745)	−0.579 (0.785)	−0.489 (0.683)	−0.118 (0.698)
Observations	1,479	1,479	1,662	1,662
Log Likelihood	−822.205	−786.682	−1,042.631	−1,027.605
Akaike Inf. Crit.	1,718.410	1,657.364	2,159.261	2,135.210
Note:				*p<0.05; **p<0.01; ***p<0.001

Supplementary Table S5. Variance Inflation Factors (VIF). The table shows the Variance Inflation Factors (VIF) for all the independent variables in the logistic regression models for the navigation performance of the participants in the Speed-race games and Least-clicks games, with and without interactions terms respectively.

	Variance Inflation Factors			
	Speed-race Games		*Least-clicks Games*	
	Without Interactions	With Interactions	Without Interactions	With Interactions
Age	1.313	1.388	1.530	1.561
Female	1.275	1.305	1.249	1.266
Asian American	1.315	1.360	1.295	1.312
African American	1.211	1.223	1.185	1.195
Foreign Language (Native)	1.467	1.457	1.322	1.332
Foreign Language	1.381	1.399	1.310	1.329
Liberal	1.235	1.316	1.224	1.253
Agreeableness	1.100	1.117	1.135	1.159
Conscientiousness	1.128	1.154	1.119	1.140
Extroversion	1.163	1.169	1.192	1.207
Neuroticism	1.176	1.178	1.074	1.099
Openness	1.111	1.155	1.150	1.150
Wikipedia_1	1.302	1.399	1.348	1.342
Wikipedia_2	1.121	1.387	1.135	1.145
Spatial_1	1.227	1.244	1.236	1.252
Spatial_2	1.147	1.172	1.148	1.155
Spatial_3	1.147	1.192	1.085	1.090
Spatial_4	1.180	1.216	1.111	1.321
Employment_1	1.381	1.584	1.311	1.512
Employment_2	1.233	1.279	1.157	1.172
Employment_3	1.150	3.662	1.165	1.193
Education_1	1.471	1.497	1.415	1.435
Education_2	1.156	1.224	1.122	1.135
Computer_1	1.363	1.430	1.394	1.425
Computer_2	1.171	1.212	1.242	1.255
Prior (Wikigame)	1.307	1.390	1.251	1.253
Prior (Source Page)	1.834	1.853	1.759	1.757
Prior (Target Page)	1.769	1.800	1.536	1.534
Order	1.038	1.043	1.014	1.016
Game Index A1	1.977	1.973	1.822	1.828
Game Index A2	2.280	2.295	2.080	2.089
Game Index B0	1.971	1.984	1.902	1.909
Game Index B1	1.943	1.939	2.040	2.042
Game Index B2	2.008	2.009	1.819	1.820
Game Index O1	1.928	1.931	1.807	1.807
Game Index O2	2.378	2.383	2.233	2.235
Foreign Language (Native):Wikipedia_2		1.499		
Liberal:Employment_3		3.953		
Wikipedia_1:Employment_1		1.185		
Wikipedia_2:Computer_2		1.155		
African American:Employment_1		1.236		
Wikipedia_2:Education_2				1.122
Spatial_4:Prior (Wikigame)				1.270
Asian American:Employment_1				1.324

Supplementary Table S6. Test result for tendency to choose Least-clicks games over Speed-race games. We tested if participants with certain characteristics are more likely to opt for Speed-race games or Least-clicks games. For binary individual characteristics variables, a Pearson's chi-squared test was conducted; for continuous characteristics variables, a Student's t-test was conducted. Least-clicks games and Speed-race games were encoded as value 1 and 0 in the calculation.

	chi2	t	pval
Female	34.40		0.00
Asian American	0.87		0.35
Foreign Language (Native)	2.63		0.11
Liberal	3.88		0.05
Spatial_1		4.32	0.00
